# Big contributions of the little brain for precision psychiatry

**DOI:** 10.3389/fpsyt.2022.1021873

**Published:** 2022-10-17

**Authors:** Sheeba Anteraper, Xavier Guell, Susan Whitfield-Gabrieli

**Affiliations:** ^1^Stephens Family Clinical Research Institute, Carle Foundation Hospital, Urbana, IL, United States; ^2^Department of Bioengineering, University of Illinois Urbana-Champaign, Urbana, IL, United States; ^3^Department of Psychiatry, Massachusetts General Hospital and Harvard Medical School, Boston, MA, United States; ^4^Department of Neurology, Massachusetts General Hospital and Harvard Medical School, Boston, MA, United States; ^5^Department of Psychology, Northeastern University, Boston, MA, United States

**Keywords:** cerebellum, psychotic disorder, functional connectivity, dentate nuclei, cerebello-thalamo-cortical circuitry

## Abstract

Our previous work using 3T functional Magnetic Resonance Imaging (fMRI) parcellated the human dentate nuclei (DN), the primary output of the cerebellum, to three distinct functional zones each contributing uniquely to default-mode, salience-motor, and visual brain networks. In this perspective piece, we highlight the possibility to target specific functional territories within the cerebellum using non-invasive brain stimulation, potentially leading to the refinement of cerebellar-based therapeutics for precision psychiatry. Significant knowledge gap exists in our functional understanding of cerebellar systems. Intervening early, gauging severity of illness, developing intervention strategies and assessing treatment response, are all dependent on our understanding of the cerebello-cerebral networks underlying the pathology of psychotic disorders. A promising yet under-examined avenue for biomarker discovery is disruptions in cerebellar output circuitry. This is primarily because most 3T MRI studies in the past had to exclude cerebellum from the field of view due to limitations in spatiotemporal resolutions. Using recent technological advances in 7T MRI (e.g., parallel transmit head coils) to identify functional territories of the DN, with a focus on dentato-cerebello-thalamo-cortical (CTC) circuitry can lead to better characterization of brain-behavioral correlations and assessments of co-morbidities. Such an improved mechanistic understanding of psychiatric illnesses can reveal aspects of CTC circuitry that can aid in neuroprognosis, identification of subtypes, and generate testable hypothesis for future studies.

## Introduction

Cerebellum's role in schizophrenia, led by Andreasen's work on the role of cerebello-thalamo-cortical (CTC) circuitry [“cognitive dysmetria” hypothesis (1, 2)], following Schmahmann's “dysmetria of thought” theory (3) is now widely acknowledged. Functional connectivity (FC) abnormalities have been reported in first-episode schizophrenia (4) and in individuals at clinical high risk (CHR) for psychosis (5–15). In spite of this mounting evidence, the FC of the dentate nuclei (DN), the primary “door-out” of the cerebellum is yet to be systematically investigated in psychotic disorders. The DN, clusters of neuronal bodies embedded in the white matter (WM) of the cerebellum (16), link the cerebellar cortex to the extracerebellar regions, and contributes to the modulation of many aspects of motor and non-motor behavior (17). However, significant knowledge gaps exist in our functional understanding of cerebellar systems (18) in imaging-based systems neuroscience. This can be attributed to spatiotemporal limitations in functional MRI, because prior to the advent of simultaneous multi-slice imaging (19), cerebellum was often excluded from the field of view. It remains to be established whether abnormalities in dentato-cerebellar functional connectivity (FC) precede the manifestation of symptoms in psychiatric diseases. Critically, we lack an effective predictive model of disease onset/progression that takes the dentato-CTC FC into account.

A recent study (20), acknowledged as a pivotal work (21), provides causal evidence for cerebellar dysfunction in schizophrenia. The basis of this advancement is on the premise that greater network-wide modulation could be achieved with cerebellar stimulation compared to cerebral cortical stimulation (22). A large and expanding body of evidence from tract tracing studies in rodents and monkeys (23–26) has revealed DN-thalamic and DN-cerebellar anatomical relations. To date, no study has comprehensively characterized dentato-CTC FC networks in humans. A further refined mechanistic picture underlying the development, regulation, and modulation of behaviors characterizing the pathophysiology in psychiatric and neurological diseases can be gained by utilizing a circuit level approach including the DN in cerebellar-focused investigations. In this perspective piece, we highlight some of the ways to go about *gaining a refined mechanistic understanding of CTC circuity* and *improving our causal understanding of symptom amelioration in treatment strategies such as transcranial magnetic stimulation (TMS)*.

### Probing dentato-CTC connectivity

**Figure 1:**
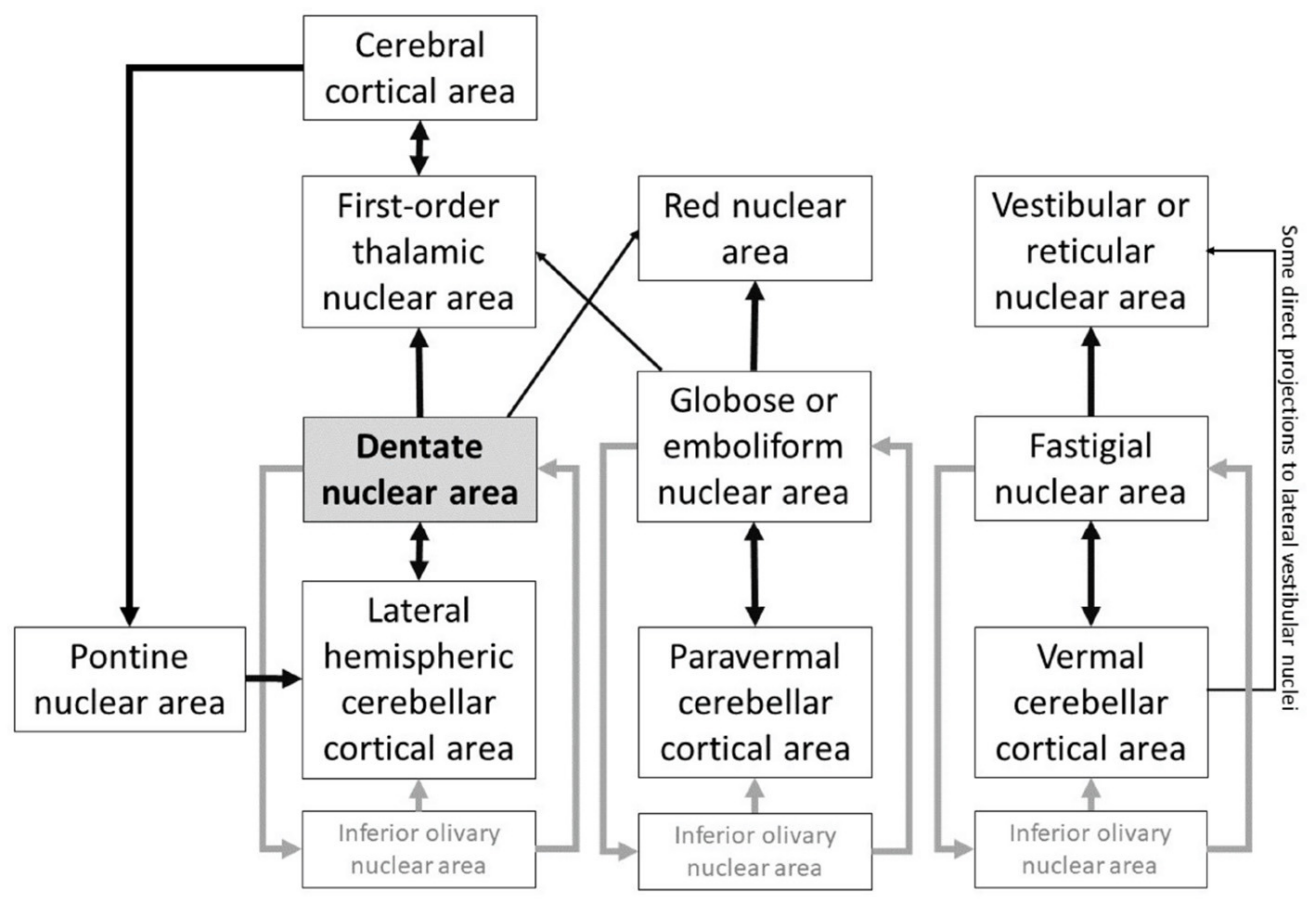
DN plays a central node in the cerebellar connections to other extracerebellar areas, and is part of a highly complex system of reverberating connections linking the nuclei of the cerebellum to the rest of the brain. Based on (28, 30, 34–40).

Heterogeneous FC arrangement of the cerebellar cortex with extracerebellar structures emerges from the backdrop of a homogenous cerebellar cortical cytoarchitecture. Anatomical connections between cerebellar cortex and extracerebellar territories engaged in cognition and affect form the basis of the neuroscience of cerebellar behavioral neurology and psychiatry (27). Improved understanding of the functional anatomy of DN provides a novel avenue to study CTC circuitry. The deep cerebellar nuclei have reciprocal connections with cerebellar cortical areas (28). The thalamic nuclei, that are anatomically linked with virtually all macroscale networks of brain organization, including dopaminergic pathways (29), receive connections from the DN and also have reciprocal projections with specific cerebral cortical areas (30). For example, DN stimulation modulates prefrontal dopamine (31), a neurotransmitter system implicated in working memory (32). Pontine nuclei receive connections from each cerebral cortical territory targeted by the DN (33), and serves as a “door-in” to the cerebellar cortex (34). These reverberating connections that link the dentate nuclei to the rest of the brain are part of the complex circuitry of the nuclei of the cerebellum (Figure 1), and establish the significance of the DN in cerebello-cerebral interactions.

### Characterization of functional territories in dentate nuclei in early psychosis

About one-third of individuals with clinical high-risk (CHR) for psychosis develop psychotic symptoms later on. For the reliable implementation of cerebellar-based therapeutics, a thorough understanding of *dentate-cerebello-cerebral* FC is imperative. DN, the largest and most lateral structure of the cerebellar nuclei system, receive projections from all aspects of the cerebellar cortex lateral to the paravermis (35). DN projects mainly to thalamus, connecting cerebellar cortex to thalamo-cortical projections, thus playing a central role in CTC circuitry. Multiple reverberating patterns exist in the connectivity between cerebellar cortex, cerebellar nuclei, and extracerebellar structures (Figure 1). These anatomical circuits establish DN as a central node in the cerebellar output circuitry, with functional specialization spanning the whole spectrum of primary, task-positive, and task-negative domains of brain function (41). The functional specialization in the DN echoes a similar set of macroscale divisions as that of the cerebral cortex. Default-mode processing [functional territory 1, FT1, in (41)] is the apex of the central axis of brain organization. Salience processing (FT2) is the cognitive opposite pole of default-mode processing, and is linked to sensorimotor control in the brain. Visual processing (FT3) is the unimodal opposite pole of sensorimotor function, and represents the third and last central component of human DN specialization. This continuous unimodal-to-transmodal view is not only a theoretical construct of cognitive science, but also an anatomical reality in the cerebral cortex (42).

**Figure 2:**
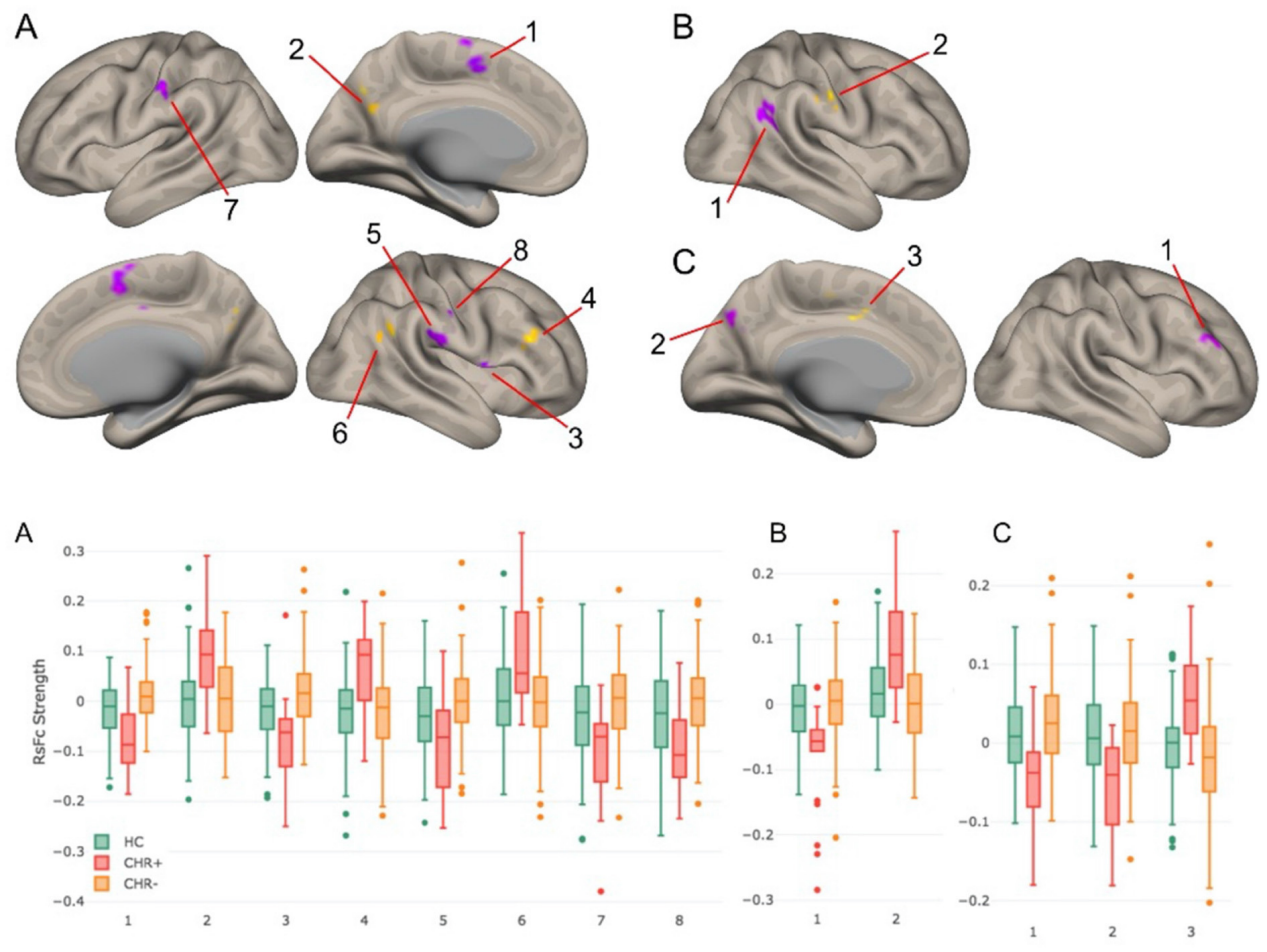
**Top panel**: RsFc results for CHR+ vs. CHR– contrast at a voxel-level height threshold of *p* < 0.005 (2-sided) and cluster size FDR corrected threshold of *p* < 0.05. **(A–C)** correspond to DMN, salience-motor, and visual functional territories of DN. **Bottom panel**: Bar plots for each of the significant clusters in the healthy control, CHR+, and CHR– groups. Notably, for each of these group contrasts, CHR– participants were not significantly different compared to healthy controls (43).

*The functional parcellation of DN (41)can add precision to the selection of seed regions of interest in studies of psychotic disorders* (43, 44). In Anteraper et al. (43), we analyzed 153 participants with CHR and 93 age-, sex-, and education-matched healthy controls (HC) in the Shanghai At Risk for Psychosis (SHARP) program. Twenty-three subjects converted to psychosis (CHR+) before the next clinical follow-up, a year later. There were no significant differences in baseline Structured Interview of Psychosis-risk Syndromes (SIPS) scores in CHR+ compared to those who did not develop psychosis (CHR-). While functional abnormalities were detected in all FTs of the DN, the DMN territory revealed more statistically significant differences compared to FT2 and FT3. Lack of anti-correlations between FT1 and DLPFC (cluster 4 in Figure 2) may indicate difficulties in executive control (43). Xie et al. (44) studied 92 patients and 86 controls, and reported that dentato-cerebello-cerebral FC abnormalities may contribute to schizophrenia symptoms and its pathophysiology.

### Improvement of our causal understanding of symptom amelioration in treatment strategies such as TMS

Precision medicine relies on the degree of our causal understanding of response to treatment. The *prediction accuracy* of *disease status/sub-types* can accelerate progress in this domain. Prediction of disease status prior to symptom manifestation (*early detection*) is another key component of this. Specific examples of how functional changes in brain network organization can be used for prediction has been reported previously in the context of conversion to psychosis (45). When combined with longitudinal behavioral measures, FC measures are emerging as promising biomarkers to understand vulnerability to predict clinical outcome in the prodromal stage of schizophrenia (46). Since the cerebellum is capable of operating in a compensatory role to restore function in response to insult, building on and leveraging non-invasive cerebellar-centric neuromodulation strategies (20) can gather a refined mechanistic understanding of brain response mechanisms in psychosis. This can aid in the development of personalized treatment approaches. DN stimulation may be achieved with TMS, although TMS will necessarily induce stimulation in the areas of cerebellar cortex located in between the stimulator and the DN, and will not achieve levels of spatial precision required to stimulate sub-regions of the DN. These two limitations, namely, the inability to avoid the cerebellar cortical surface, and the inability to stimulate specific DN sub-regions, may be both overcome by two emerging methods of non-invasive stimulation. Low-intensity focused ultrasound has shown spatially precise modulation of brain activity in deep brain regions located beneath the cerebral cortex, such as the amygdala (47). Temporally Interfering Electric Fields, another emerging method of non-invasive brain stimulation, has shown successful modulation of deep brain territories such as the hippocampus without affecting neighboring lateral or surface areas (48).

## Future directions

Building upon our work, we envision that further exploration of the cerebellar output circuitry will generate valuable contributions to the field of translational and precision psychiatry. Novel gradient-based analysis strategies (49) can be used to complement existing brain mapping approaches (50), and to detect functional abnormalities in psychiatric disorders that may remain hidden using other methods of analysis (51, 52). Cerebellum's interplay with cerebral cortical dynamics is still poorly understood. New results have demonstrated that blocking/stimulating cerebellar cortical output through DN *via* the thalamus (CTC pathway) can modulate cerebral cortical dynamics as demonstrated by suppression/triggering of movement initiation (53). Extensive disruptions in CTC connectivity has been linked to “cognitive dysmetria” (1, 2) and increased risks for psychosis conversion (6, 7). Future research might investigate the possibility that some of these alterations may be specific to particular psychiatric disorders, while others may be linked to broader domains of psychopathology [as in (51)]. We recently estimated effective connectivity using spectral dynamic causal modeling (DCM) (54) in the Human Connectome Project dataset to examine cerebello-cerebral interactions indexed by FC between the cerebellar and cerebral cortex (55). This work supports the Universal Cerebellar Transform (UCT) theory, which posits that the neurological processes underlying cerebellar modulation of movement, thought and emotion (3, 56, 57) are the same. The existence of a UCT is a fundamental underpinning of the dysmetria of thought theory that may be further interrogated using DN-targeted non-invasive stimulation in the future. Lastly, emerging methods of non-invasive brain stimulation may allow the development of spatially precise targets within DN as tools for the treatment of disease and for the study of cerebellar functional anatomy.

## Technological advances in MRI

Blood oxygenation level dependent (BOLD) MRI contrast to noise (CNR) ratio, which is directly proportional to temporal signal-to-noise ratio (tSNR), is remarkably better at ultra-high field strengths. When used in combination with parallel transmit head coils (58) and optimized pulse sequences (59), 7T can offer unprecedented improvements in tSNR and spatiotemporal resolution for fMRI (60). Superior BOLD CNR that comes with these technological advances can be used for investigating the full range of DN-cerebral, DN-thalamic and DN-cerebellar FC. The field of cerebellar functional neuroanatomy is emerging with novel theories, which include functional gradients that dictate the position and relationship between cerebellar FTs. Identifying FTs of the DN with 7T resting-state fMRI and using these functional parcels to better characterize functional abnormalities in cerebellar-linked neuropathology can thus generate valuable contributions to the field of cerebellar neuroscience and translational psychiatry.

## Data availability statement

The original contributions presented in the study are included in the article/supplementary material, further inquiries can be directed to the corresponding author.

## Ethics statement

The studies involving human participants were reviewed and approved by Institutional Review Boards at Beth Israel Deaconess Medical Center and also by the Shanghai Mental Health Center. Written informed consent to participate in this study was not obtained since no human studies are present.

## Author contributions

SA conceived the presented idea. SA and XG performed the data analysis and verified the analytical methods for the two publications that are highlighted in this perspective piece. SW-G contributed the data needed for the work and fueled the critical thinking needed to accomplish the work. All authors discussed the results and contributed to the final manuscript. All authors contributed to the article and approved the submitted version.

## Conflict of interest

The authors declare that the research was conducted in the absence of any commercial or financial relationships that could be construed as a potential conflict of interest.

## Publisher's note

All claims expressed in this article are solely those of the authors and do not necessarily represent those of their affiliated organizations, or those of the publisher, the editors and the reviewers. Any product that may be evaluated in this article, or claim that may be made by its manufacturer, is not guaranteed or endorsed by the publisher.
